# Correction: Ku-0063794 is a specific inhibitor of the mammalian target of rapamycin (mTOR)

**DOI:** 10.1042/BJ20090489_COR

**Published:** 2026-10-14

**Authors:** Juan M. García-Martínez, Jennifer Moran, Rosemary G. Clarke, Alex Gray, Sabina C. Cosulich, Christine M. Chresta, Dario R. Alessi

**Affiliations:** 1 MRC Protein Phosphorylation Unit, College of Life Sciences, University of Dundee, Dow Street, Dundee DD1 5EH, Scotland, U.K.; 2 Division of Signal Transduction Therapy, College of Life Sciences, University of Dundee, Dow Street, Dundee DD1 5EH, Scotland, U.K.; 3 Division of Cell Biology and Immunology, College of Life Sciences, University of Dundee, Dow Street, Dundee DD1 5EH, Scotland, U.K.; 4 Division of Molecular Physiology, College of Life Sciences, University of Dundee, Dow Street, Dundee DD1 5EH, Scotland, U.K.; 5 Cancer Bioscience, Astrazeneca, Alderley Park, Cheshire, SK10 4TG, U.K.

**Keywords:** Akt/protein kinase B (PKB), cancer, kinase inhibitor, phosphoinositide 3-kinase (PI3K), p70 ribosomal S6 kinase (S6K), serum and glucocorticoid protein kinase (SGK)

It has come to the attention of the authors of the article “Ku-0063794 is a specific inhibitor of the mammalian target of rapamycin (mTOR)” (DOI: 10.1042/BJ20090489) that an error occurred in Figure 5A, where the Western blot representing phospho-S6K1 (Thr389) was inadvertently duplicated and used in place of the panel showing phospho-S6K1 (Thr229).

**Figure 5:**
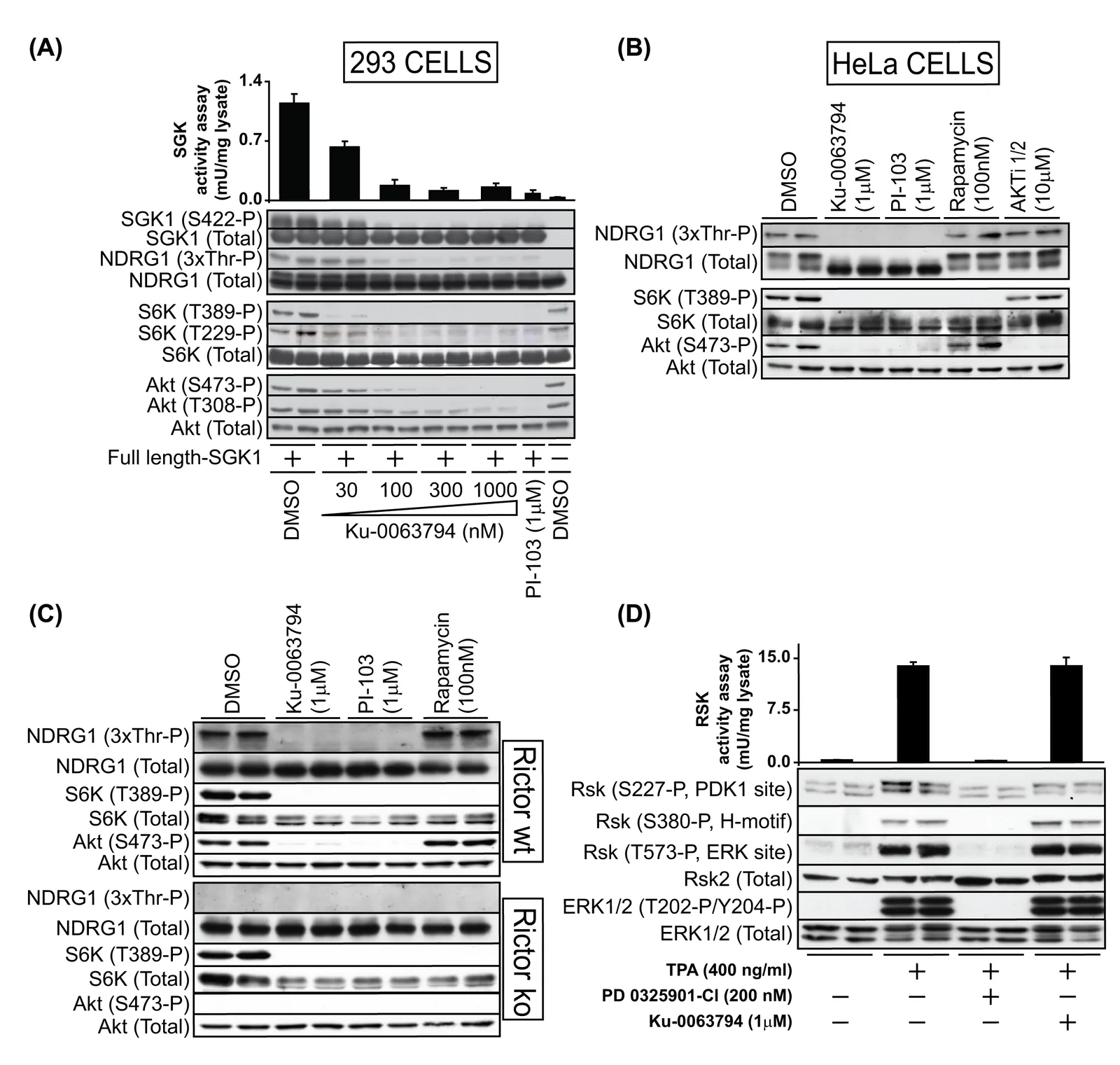
Ku-0063794 induces a marked dephosphorylation of 4E-BP1

This duplication arose unintentionally during figure assembly. The Figure has now been corrected using the original experimental data to display the correct phospho-S6K1 (Thr229) immunoblot.

The Publisher has reviewed the original data and approves the publication of a correction.

The authors confirm that this error does not affect the data interpretation or the conclusions of the study. The authors apologise for this error and any inconvenience caused.

